# Analysis of Relevant Features from Photoplethysmographic Signals for Atrial Fibrillation Classification

**DOI:** 10.3390/ijerph17020498

**Published:** 2020-01-13

**Authors:** César A. Millán, Nathalia A. Girón, Diego M. Lopez

**Affiliations:** Telematics Engineering Research Group, Telematics Department, Universidad Del Cauca (Unicauca), Popayán 190002, Colombia; cesaramillan@unicauca.edu.co (C.A.M.); nathaliag@unicauca.edu.co (N.A.G.)

**Keywords:** atrial fibrillation, AF, photoplethysmography, PPG, feature selection

## Abstract

Atrial Fibrillation (AF) is the most common cardiac arrhythmia found in clinical practice. It affects an estimated 33.5 million people, representing approximately 0.5% of the world’s population. Electrocardiogram (ECG) is the main diagnostic criterion for AF. Recently, photoplethysmography (PPG) has emerged as a simple and portable alternative for AF detection. However, it is not completely clear which are the most important features of the PPG signal to perform this process. The objective of this paper is to determine which are the most relevant features for PPG signal analysis in the detection of AF. This study is divided into two stages: (a) a systematic review carried out following the Preferred Reporting Items for a Systematic Review and Meta-analysis of Diagnostic Test Accuracy Studies (PRISMA-DTA) statement in six databases, in order to identify the features of the PPG signal reported in the literature for the detection of AF, and (b) an experimental evaluation of them, using machine learning, in order to determine which have the greatest influence on the process of detecting AF. Forty-four features were found when analyzing the signal in the time, frequency, or time–frequency domains. From those 44 features, 27 were implemented, and through machine learning, it was found that only 11 are relevant in the detection process. An algorithm was developed for the detection of AF based on these 11 features, which obtained an optimal performance in terms of sensitivity (98.43%), specificity (99.52%), and accuracy (98.97%).

## 1. Introduction

This research is an extension of the work previously presented at the 16th International Conference on Wearable Micro and Nano Technologies for Personalized Health, p-Health 2019: Systematic Review on Features Extracted from PPG Signals for the Detection of Atrial Fibrillation [1], which made it possible to obtain the features reported in the literature that have been used for the detection of atrial fibrillation (AF). Therefore, in this paper, different features and AF classifiers are experimentally evaluated to determine which of them achieves the best performance in terms of sensitivity, specificity, and accuracy.

Atrial fibrillation is the most prevalent cardiac arrhythmia in clinical practice. It has a significant impact on morbidity, mortality, and decreased quality of life, besides being a major cause of health care expenditure [2]. It is estimated that AF affects about 33.5 million people, representing approximately 0.5% of the world’s population, and it is forecasted that annually 5 million new cases will be found [3]. However, since atrial fibrillation is usually clinically silent, i.e., asymptomatic, it is not diagnosed, or it is diagnosed so late that the condition has already led to different health problems.

Commonly, AF screening is only recommended in patients older than 65 years, due to the high prevalence of this cardiac arrhythmia from this age. Screening is usually carried out through techniques such as pulse palpation but, despite being highly recommended and cost-effective method, low percentages of specificity have been reported when using it (72%). Therefore, this technique must be combined with more expensive technologies such as 12-lead electrocardiography (ECG) and/or 24- or 48-hour Holter monitoring for an accurate diagnosis. Currently, electrocardiogram (ECG) analysis is the main diagnostic criteria for AF. It is based on signal analysis, which shows the series of waves that are related to the electrical impulses that occur during each heartbeat [4].

In order to achieve early and opportune detection of such cardiac arrhythmia, over the years, several methods have been developed with diverse approaches, most notably the ones using photoplethysmography (PPG) sensors. PPG makes the screening process more accessible as well as more opportune and allows performing it on patients of different ages and not only in those with high-risk factors. PPG is an optical technology that can detect changes in blood flow during heart activities and has traditionally been used to measure oxygen saturation and heart rate. Currently, it is also used to build the heart rhythm signal from it. Compared to electrocardiogram procedures, obtaining PPG signals is much easier and more comfortable, as it can be measured at the fingertips, wrists, or earlobes with simple, portable devices. Therefore, PPG signal analysis is considered useful for detecting and identifying patients with AF.

In order to detect atrial fibrillation in cardiac rhythm signals, the latter must be digitally processed, allowing to model, analyze, characterize, and understand the activity of the cardiac muscle, which controls the initiation, maintenance, and termination of AF episodes. This processing is commonly focused on the extraction and analysis of the different features of the signal. However, given the novelty of using PPG signals for AF detection, it is not completely clear which are the most relevant features of the signal to perform the diagnosis. The objective of this article is to evaluate the features extracted from the PPG signal reported in the literature for the detection of atrial fibrillation, and subsequently, through a processing algorithm and machine learning, to determine which of the features achieves the best performance for the classification of AF.

## 2. Materials and Methods

This study was divided into two stages: first, a systematic review was carried out, following the PRISMA-DTA statement in six databases, to identify the reported features extracted from the PPG signal for the detection of AF, and second, an evaluation of them was performed, using machine learning to determine which of the features achieves the best performance in classification of AF.

### 2.1. Systematic Review

A systematic review related to mobile systems for detecting atrial fibrillation using photoplethysmography was conducted in six databases (Pubmed, Science Direct, Scopus, IEEE Xplore, Engineering Village, and Mendeley), following the PRISMA-DTA methodology. Furthermore, an analysis of the reference lists of the articles found was performed. The search was carried out between July and October 2018. The search chain used in the databases was constructed around three groups of keywords: terms used in the literature alluding to detection (detection, diagnosis, classification, screening), device used for data collection (smartphone, wristband, wearable, activity monitor), and used measures (PPG, photoplethysmography, accelerometer), combining them with the keyword: Atrial Fibrillation.

The following selection criteria were established. First, only articles published in English or Spanish were included. Second, publication date from 2008. Third, the system presented in the article must use photoplethysmography for the detection of AF. Fourth, the system had to be mobile, which means that it had to use wearable sensors and wireless technologies. Lastly, AF detection had to be based on the extraction and analysis of heart rhythm signal features.

In order to determine the inclusion criteria, the title and abstract were taken into account. Thus, excluding those which at the time of this initial review did not fulfill the established selection criteria. Subsequently, potentially eligible articles were selected for full reading, and their inclusion or exclusion was then determined. Each article was reviewed, and its essential aspects synthesized. These were: features used for AF detection, classification of these features, and reported results of sensitivity, specificity, and accuracy.

### 2.2. Experimental Evaluation of the Features

**Figure 1:**
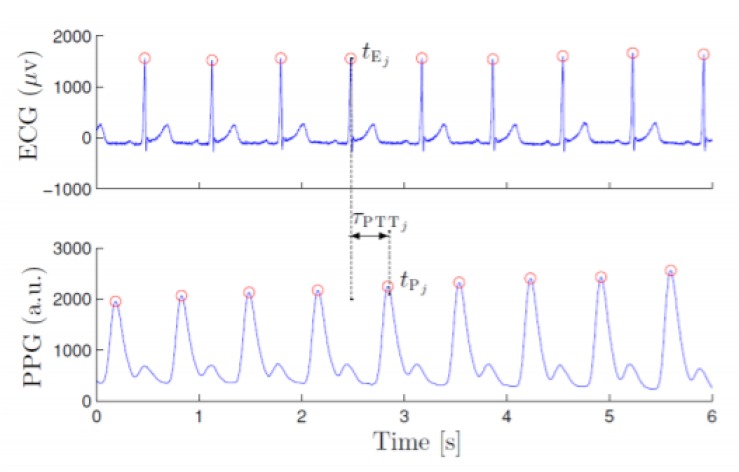
Comparison between ECG and PPG signals [11].

Once the features found in the literature and the results reported for them were extracted, the development of an algorithm in Python was carried out following the intermediate phases of the Cross-Industry Standard Process for Data Mining—CRISP-DM methodology (data preparation, modeling, and evaluation). This algorithm has three functions: to extract the features employing digital processing of cardiac rhythm signals, to select the most determining features employing machine learning, and to evaluate the performance presented by different groups of these features in the detection of AF. The algorithm is based on the analysis of the interbeat intervals, which can be observed and analyzed in both ECG and PPG signals. Due to the lack of public datasets of heart rhythm signals taken through photoplethysmography sensors from patients diagnosed with atrial fibrillation, the algorithm developed for the digital processing of heart rhythm signals and subsequent detection of AF was implemented based on ECG signals found in five open Physionet datasets (1656 signals: 828 normal sinus rhythm signals and 828 signals with presence of atrial fibrillation) [5,6,7,8,9]. This was possible because the PPG and ECG signals are closely related. These signals only differ in a constant delay between the peak of the ECG signal and the peak of the PPG signal, commonly known as pulse transition time (PPT), as shown in Figure 1. This is caused due to the time elapsed from the occurrence of ventricular systole until the PPG sensor detects the event. It should be noted that beyond this remarkably constant time delay, there is no major discrepancy between the intervals between peaks. This was demonstrated in [10], indicating that the correlation index between the two intervals is very close to 1. This leads to the conclusion that the extraction of the interbeat intervals for the detection of atrial fibrillation can be performed regardless of whether it is an ECG or PPG signal. Due to the retrospective nature of this records-based research study, which is using only publicly available and de-identified physiological databases found in Physionet, the study is exempt from approval from an ethics committee, according to the Common Rule (45 CFR 46) of the U.S. Department of Health & Human Services.

### 2.3. Three Stages of the Implemented Algorithm

#### 2.3.1. Stage 1: Pre-Processing

The quality of an algorithm for signal processing depends significantly on the quality of the signal used as the input: When the quality of the input signal is lower, the algorithm’s performance will be lower. Therefore, pre-processing is a crucial stage since it adapts the signal used by the algorithm. In this case, pre-processing includes a resampling process, a filtering process, and a normalization process. The resampling is based on Newton’s general interpolation, the filtering is performed using Butterworth’s band-pass filter, and the normalization is based on the 97th percentile of the heart rhythm signal.

#### 2.3.2. Stage 2: Processing

The systematic review allowed determining that the features extracted from the PPG signal could be classified according to the domain the signal belonged to at the time of its analysis. This way, the features were classified into: features in the time domain, features in the frequency domain, and features in the time-frequency domain. Based on the results of sensitivity, specificity, and accuracy reported in the different studies, the feature selection process was carried out manually, giving it greater relevance and choosing those features that reported higher values in the mentioned results. Thus, in order to distinguish between signals with the presence of atrial fibrillation and signals with normal sinus rhythm, 27 signal features were extracted and analyzed. The analysis is performed to signals of one-minute duration following the recommendation of the cardiologist supporting this study who stated that this time was enough to detect atrial fibrillation.

##### Features Extraction

Time-Domain Features Extraction:

Thirteen features were extracted in the time domain. For this extraction, the peaks of the heart rhythm signal were detected. Due to the pre-processing stage, it was possible to implement a simple algorithm to detect all peaks in the PPG signal above an 88 percentile and to calculate just the intervals between those peaks. From obtaining the intervals between the peaks, also known as interbeat intervals (IBI), the following features were extracted: root mean square of successive differences (RMSSD), sample entropy (SampEn), coefficient of sample entropy (CosEn), Shannon entropy (ShE), the standard deviation of Poincaré plot perpendicular to the line-of-identity (SD1), the standard deviation of the Poincaré plot along the line-of-identity (SD2), the standard deviation of R-R intervals (SDRR), ratio of SD1/SD2 (SD1/SD2), total HRV (S), mean, standard deviation (STD), mean absolute deviation (MeanAD), and median absolute deviation (MAD).

Frequency Domain Features Extraction:

Eight features were extracted in the frequency domain. It was necessary to use the fast Fourier transform (FFT) to convert the signal from the time domain where it was recorded to the frequency domain, and then proceed to perform the feature extraction. In this domain, the heart rhythm signal can be separated into its component rhythms (ultra-low frequency—ULF; very-low frequency—VLF; low-frequency—LF; and high frequency—HF), which operate in different frequency ranges and provide additional relevant information to distinguish an Normal Sinus Rhythm (NSR) signal from an AF signal. In this analysis, the extracted features were: max peak, summed spectral energy (TPW), spectral entropy (SpEn), VLF, LF, HF, mean, and standard deviation (STD).

Time-Frequency Domain Features Extraction:

Six features were extracted in the time-frequency domain. By transforming the signals from the original time domain to the time-frequency domain, it is possible to observe the variability in the power of the different frequencies over time. The wavelet transform was used to perform this transformation, from which we obtained the approximation coefficients (cA) and detail coefficients (cD) and extracted the following features: average energy (AEcA, AEcD), mean absolute value (MAVcA, MAVcD), and standard deviation (STDcA, STDcD).

Attribute Selection:

Once all the features were extracted from the signal, a selection was carried out to determine which features are decisive in the detection of atrial fibrillation. The process for performing this selection is presented below:

Selection: All the features extracted from the signal in the time, frequency, and time–frequency domain were included. In total, 27 features were taken into account.

Structuring: A dataset was built formed by a 1656 × 28 matrix, where the 1656 rows represent the number of signals used for training, showing the results obtained for each one of the features of the heart rhythm signals divided into two groups: 828 normal sinus rhythm signals and 828 signals with presence of atrial fibrillation. The 28 columns represent each one of the features previously described, and the last column represents the target variable (Vt), i.e., the attribute to be predicted or modeled. In this case, the target variable is qualitative. It indicates the presence or absence of atrial fibrillation, and it is marked with 1 for presence and 0 for absence.

In order to conduct the selection process of the most relevant features, 27 different algorithms were created from the combination of the evaluators, classifiers, and search methods available in WEKA, version 3.8.2 (University of Waikato, Hamilton, New Zeland). Thereby, ten evaluators, four classifiers, and three different search methods were used. It should be noted that all available WEKA methods have been deployed. The evaluators, classifiers, and search methods used were:

- Evaluators: CfsSubsetEval (E1), ClassifierAttributeEval (E2), ClassifierSubsetEval (E3), CorrelationAttributeEval (E4), GainRatioAttributeEval (E5), InfoGainAttributeEval (E6), OneRAttributeEval (E7), ReliefFAttributeEval (E8), SymmetricalUncertAttributeEval (E9), WrapperSubsetEval (E10).
- Classifiers: DecisionTable (C1), JRip (C2), OneR (C3), PART (C4).
- Search methods: BestFirst (M1), GreedyStepwise (M2), Ranker (M3).

This way, the different algorithms created to make the selection of attributes are described in Table 1.

Features grouping: Lastly, to determine which features had the most significant influence on the AF classification, the obtained results from the algorithms created for the selection were analyzed. The features grouping was performed according to the way each of the algorithms presented the results. Some of them organize all the features ranked from the most relevant to the least relevant. Others select from the whole group of features a subset of them, which are considered to be the ones that influenced the AF detection. Table 2 describes the grouping of features.

This way, the following groupings of the feature were created from the analysis mentioned above:

○ Group 1 (26 + Vt): All features.
○ Group 2 (8 + Vt): Mean, MAD, RMSSD, SD1, SDRR, MAVcA, AEcA, STDcA.
○ Group 3 (11 + Vt): Group 2 + STD, S, SpEn.
○ Group 4 (15 + Vt): Group 3 + MeanAD, SD2, AEcD, STDcD.
○ Group 5 (20 + Vt): Group 4 + SampEn, CosEn, She, MaxPeak, MAVcD.

#### 2.3.3. Stage 3: Classification Algorithm

As a third stage, a machine learning model was trained, whose function is to classify the input signal as a signal with the presence or absence of atrial fibrillation to evaluate the performance in AF detection from 5 different datasets based on the groups of features described above. It is important to emphasize that each dataset is made up of 1656 rows (number of signals used for training) and that the number of columns corresponds to the number of features of the evaluated group (27 for group 1, 9 for group 2, 12 for group 3, 16 for group 4 and 21 for group 5).

To create the machine learning model, the Scikit-learn tool developed for Python was used, and 11 models were implemented to find the most accurate algorithm. Those supervised learning models evaluated are: XGBClassifier, KNeighborsClassifier, SVC, NuSVC, DecisionTreeClassifier, RandomForestClassifier, AdaBoostClassifier, GradientBoostingClassifier, GaussianNB, LinearDiscriminantAnalysis, and QuadraticDiscriminantAnalysis.

It is crucial to notice that the process of implementing the classification algorithm is directly related to the final stage of features selection since the models selected for the implementation of the classifying algorithm must be trained with each group of features, aiming at the analysis, evaluation, and selection of the model and the group of features that have achieved the highest performance in the classification of the test set.

### 2.4. Algorithm Evaluation

Following the CRISP-DM evaluation phase after selecting the models and the group of features that presented the best performance, the evaluation of algorithms is performed. It is worth to mention that only the reliability calculated for the models under consideration is applied, taking into account the data used for training and testing the algorithm. These results can be calculated using confusion matrices and receiver operating characteristic (ROC) curve analysis.

## 3. Results

### 3.1. Systematic Review

Initially, 224 studies were obtained as a result of the search for keywords in the databases. Based on study selection, 149 were excluded from these 224 studies, resulting in 75 articles for an in-depth review, and 15 more added from reviewing the references. From the remaining 90 articles, 70 were excluded because of not meeting the eligibility criteria. Thus, a group of 20 articles was obtained for analysis with five articles about the same system. Therefore, the total number of articles was finally reduced to 16.

Table 3 presents the features extracted from the PPG signals found in the literature. These features are classified into three categories according to the extraction process performed: with the signal in the time domain, the signal in the frequency domain, or the signal in the time-frequency domain. Its acronym and its respective identifier accompany each feature.

Table 4 shows a comparison of the studies found in the systematic review. The title of each study is presented, along with the features extracted from the signal, used to perform the detection of atrial fibrillation (features are classified into the three categories as mentioned above), and the reported results of sensitivity, specificity, and accuracy.

In those studies, in which different combinations of these features have been analyzed, different values of sensitivity, specificity, and accuracy are obtained according to each combination made. The results are presented as intervals, taking the minimum value obtained as the lower limit and the maximum value as the upper limit. When only the overall value obtained is reported in studies, the results of sensitivity, specificity, and accuracy are presented as a single value.

### 3.2. Features Evaluation

Table 5 shows a comparison based on the accuracy results obtained by the implemented models for each one of the five groups of features.

Likewise, Table 6 represents a comparison based on the analysis of descriptive statistical data, extracted from the results obtained in the previous table.

Based on the results of accuracy obtained by the models for AF detection, the main objective of the attribute selection process is to select the smallest subset of attributes. Also, taking into consideration that the percentage of classification is not significantly affected and that the resulting distribution is as similar as possible to the original one, the classifiers with the highest minimum percentage of accuracy and the group with the least number of features that obtained the best results were chosen. These are:

1. XGBClassifier, AdaBoostClassifier: achieved consistent performance and a minimum accuracy value of 98.55% in both cases, compared to the detection of AF in the heart rhythm signals belonging to the test set.
2. Group 3, being the smallest group of features (11) and maintaining or improving the percentage of accuracy that the total group of features (Group 1) obtains in the detection of AF.

### 3.3. Algorithm Evaluation

The results obtained when running the XGBClassifier and AdaBoostClassifier models with Group 3 of the features are presented in Table 7.

**Figure 2:**
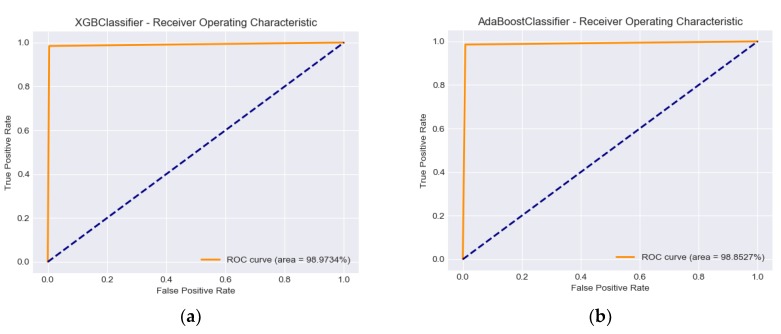
(**a**) XGBClassifier ROC Curve and AUC; (**b**) AdaBoostClassifier ROC Curve and AUC.

Figure 2 demonstrates the performance of the classification models using the ROC score and the related aggregate measure of performance AUC (area under the ROC curve) at all classification thresholds.

## 4. Discussion

To our knowledge, the original article on which this study is based is the first publication of a systematic review about the extraction and analysis of features from PPG heart rhythm signals for the detection of atrial fibrillation. It is also the first study evaluating the performance reported in studies and adapting the PRISMA extension for systematic reviews on the Diagnostic Test Accuracy (PRISMA DTA). This systematic review made it possible to identify and classify the features of the PPG heart rhythm signal commonly used for the detection of atrial fibrillation, and to select those having achieved the best performance. From the analysis of these selected characteristics deploying machine learning, it was possible to objectively choose those with a real influence on the detection process of atrial fibrillation. The resulting models have been implemented, finally obtaining an algorithm for the classification of the AF cardiac arrhythmia.

Based on the process of feature extraction from the signal, our study demonstrates that PPG signals can be classified depending on the domain in which the signal is analyzed for feature extraction. Thus, identifying three categories: features in the time domain, features in the frequency domain and features in the time-frequency domain. Furthermore, it was found that for feature extraction and subsequent detection of atrial fibrillation, signal analysis prevails exclusively in the time domain with 12 articles (75% of the articles analyzed). However, four of the studies performed an analysis as a complement to it. Thus, in addition to the analysis in the time domain, two studies analyzed the signal in the frequency domain, and the other two analyzed the signal in the time-frequency domain.

Forty-four features were found extracted from the heart rhythm signal for the detection of AF, out of which 29 were extracted from the signal analyzed in the time domain, 12 from the signal analyzed in the frequency domain, and three from the signal analyzed in the time-frequency domain. Two graphical methods were found based on the analysis of the distribution or dispersion of the intervals between the heartbeats: Poincaré Plot and Scattergram Plot. It was also found that the algorithms developed for the detection of AF use a variable number of those features. Thereby, they not only use them individually but also group them in as many combinations as possible for obtaining better results. The features with the highest prevalence of use among all the articles analyzed were: Shannon entropy, root mean square of successive differences and its normalized signal, sample entropy, and standard deviation, used in more than 50% of the studies.

Based on the reported results of sensitivity, specificity, and accuracy, it was found that these values in the studies performing analyses in the time domain were in the ranges: 69–100%, 44.2–97.65%, and 68.4–98.1% respectively. For the two studies that performed an analysis in the frequency domain complementary to the analysis in the time domain, the results of sensitivity, specificity, and accuracy were in the ranges: 85–100%, 88.7–100%, and 95.9–100% respectively. The two studies that performed an analysis in the time–frequency domain complementary to the analysis in the time domain reported only the results of accuracy, which varied between 91.8% and 99.4%.

These findings allow us to conclude that when performing a complementary analysis to the analysis of the signal in the time domain, the results are improved. The performance improvement of AF detection algorithms when analyzing the signal in the frequency and time–frequency domain is achieved because the variability in power of the different frequencies over time can be observed with greater precision in these domains. Hence, the heartbeats in atrial fibrillation are irregularly irregular. Shashikumar [14] explained this with the fact that normal PPG signals will show dominant frequencies in very low frequencies (1–2 Hz). In contrast, in AF signals, there will no longer be any dominant frequency due to the irregular rhythm of the heartbeats. The presented results suggest future methods in the detection of atrial fibrillation. The analysis of the signal should be performed not only in the time domain but also in the frequency and time-frequency domain.

A limitation of the systematic review reported in the original article was that the performance results in detecting atrial fibrillation in terms of sensitivity, specificity, and accuracy reported in the literature were not fully comparable because most studies used different datasets for testing. For overcoming this limitation, an experimental evaluation of the different features found was performed. From the 44 features identified in the review, 27 were implemented and evaluated using two different types of machine learning algorithms created in WEKA from the available evaluators, classifiers, and search methods. The first ranked features and the second selected a small group of features based on their incidence against the target variable. Subsequently, the features were ordered manually according to the results obtained, giving greater importance to those chosen by the algorithms as more incidents, obtaining five different groups of features. From this, it was found that only 11 features (Group 5) have a real influence on the detection process. These features were: Mean, MAD, RMSSD, SD1, SDRR, MAVcA, AEcA, STDcA, STD, S, and SpEn, which formed a dataset for the evaluation of the final atrial fibrillation detection algorithm. The machine-learning algorithm was implemented for the detection of AF based on these 11 features, which resulted in optimal performance in terms of sensitivity (98.43%), specificity (99.52%), and accuracy (98.97%).

The obtained results, when evaluating the different models with each one of the five groups of features, allowed to demonstrate that the accuracy values of the majority of the models were considerably high, exceeding 90%, except for the SVC and NuSVC models, which obtained values between 50% and 76%.

This means that the performance obtained by integrating the different stages of the proposed algorithm for the detection of atrial fibrillation can be evidenced by the results obtained in terms of sensitivity, specificity, and accuracy when evaluating the XGVClassifier and AdaBoostClassifier models with the features group mentioned above. Sensitivity represents the ability of the algorithms to correctly determine AF cases, obtaining results of 98.43% and 98.55%. Specificity indicates the ability of the algorithms to correctly determine non-AF cases, obtaining results of 99.52% and 99.15%. Accuracy represents the ability of the algorithms to correctly differentiate FA and non-AF cases, obtaining results of 98.97% and 99.15%.

In the development and implementation of software systems involving different stages, it is crucial to test not only the performance of each one of the stages, but also the performance presented in the integration of the entire system. Through this process, it can be evidenced if the developed system fulfills or not the expected results of the project. For carrying out a correct evaluation of the extracted features, the data mining criteria were taken into account since they are more specific and precise. For this reason, the objective basis on which the evaluation is justified is the statistical indicators obtained when executing the models.

Finally, compared to the articles found in the literature and included in the systematic review, in which the extraction of features through the analysis of the heart rhythm signal is performed taking into account the combination of the time domain and either the frequency or time-frequency domain, the presented study conducts the feature extraction by analyzing the heart rhythm signal on the three domains. This led to selecting the smallest group of features in order not to include those that did not influence AF detection, achieving optimal results in terms of sensitivity, specificity, and accuracy.

## 5. Conclusions

Technological developments in biomedical sensors and wireless techniques have led to the emergence of solutions for different challenges in the field of m-Health. The systematic review made it possible to identify the tendencies followed in systems for detecting atrial fibrillation. Thereby, an in-depth analysis of the features extracted from the heart rhythm signal used for the detection of this arrhythmia was carried out. Through this analysis, it was possible to identify and select those features that presented the best results in terms of sensitivity, specificity, and accuracy. This allowed for the definition of those determinants in the process of detecting AF.

The attribute selection process was carried out using machine learning algorithms such as evaluators and classifiers. Hence, it was possible to discard irrelevant information and features in order to improve the performance of the developing algorithm. Furthermore, it allows selecting the smallest subset of attributes without significantly affecting the classification percentage and obtaining a distribution as similar to the original as possible.

In the literature review, it was found a clear tendency to analyze the heart rhythm signal only in the time domain for feature extraction. Nevertheless, it was evident that the best results reported in the literature were obtained by analyzing the signal in the time domain, and additionally in the frequency or time-frequency domain. However, in the selection and evaluation of features, it was found that from those features extracted in the frequency domain, only one had real incidence in the process of detection of atrial fibrillation and that from those extracted in the time-frequency domain, three influenced. Likewise, a lack of investigation and implementation of features in these two domains was evident. Additionally, it was not possible during the development of this project to find public databases of heart rhythm signals of patients diagnosed with atrial fibrillation taken via photoplethysmography sensors. Nevertheless, because the detection of atrial fibrillation is done through the analysis of RR intervals and the close correlation between ECG and PPG signals, it was possible to use ECG databases for the construction and training of our algorithm.

We are currently performing the implementation of a mobile system that allows the classification of atrial fibrillation through a mobile application, which performs the acquisition of the heart rhythm signal through a wearable photoplethysmography sensor. The signal is processed through the machine learning algorithm developed to evaluate the performance of both the algorithm and the mobile system in a real environment.

## Figures and Tables

**Table 1:** List of algorithms created for the attributes’ selection.

Algorithm (Id)	Evaluator	Classifier	Search Method
1	E2	C1	M3
2	E2	C2	M3
3	E2	C3	M3
4	E2	C4	M3
5	E3	C2	M1
6	E4		M3
7	E5		M3
8	E6		M3
9	E7		M3
10	E8		M3
11	E9		M3
12	E1		M1
13	E3	C1	M1
14	E3	C3	M1
15	E3	C4	M1
16	E3	C1	M2
17	E3	C2	M2
18	E3	C3	M2
19	E3	C4	M2
20	E10	C1	M1
21	E10	C2	M1
22	E10	C3	M1
23	E10	C4	M1
24	E10	C1	M2
25	E10	C2	M2
26	E10	C3	M2
27	E10	C4	M2

**Table 2:** Features grouping.

Features	Algorithm (Id)																										
	1	2	3	4	5	6	7	8	9	10	11	12	13	14	15	16	17	18	19	20	21	22	23	24	25	26	27
	Time-domain																										
Mean																											
STD																											
MeanAD																											
MAD																											
RMSSD																											
SD1																											
SD2																											
SDRR																											
S																											
SampEn																											
CosEn																											
ShE																											
	Frequency domain																										
Max Peak																											
Mean FFT																											
STD FFT																											
TPW																											
SpEn																											
LF																											
HF																											
VLF																											
	Time-Frequency domain																										
MAVcA																											
MAVcD																											
AEcA																											
AEcD																											
STDcA																											
STDcD																											
																											
		FEATURES CHOSEN BY SUBSET EVALUATORS																									
		FEATURES WITH RANKING > 0.1																									
		FEATURES WITH RANKING > 0 & < 0.1																									
		FEATURES WITH RANKING ≤ 0																									

**Table 3:** List of features extracted from the PPG signal that is used for AF detection.

Domain	ID	Features	Acronym
Time	1	Asymmetry	
	2	Average of the absolute value of the differences	
	3	Covariance	CoV
	4	Coefficient of Sample Entropy	CosEn
	5	Coefficient of variation	CV
	6	Interquartile Range	Iqr
	7	Maximum	Max
	8	Mean	M
	9	Mean Absolute Deviation	MAD
	10	Mean Absolute Error	MAE
	11	Mean Absolute Percentage Error	MAPE
	12	Mean Error	ME
	13	Median	MED
	14	Median Peak Height Rise	mPHR
	15	Minimum	Min
	16	Normalized Absolute Deviation	NADev
	17	Normalized Absolute Difference	NADiff
	18	Normalized RMSSD	nRMSSD
	19	Percentage of interval differences of successive intervals	pNNx
	20	Probability Density Function	PDF
	21	Reliability	
	22	Robust standard deviation	STD
	23	Root Mean Square Error	RMSE
	24	Root Mean Square of Successive Differences	RMSSD
	25	Sample Entropy	SampEn
	26	Shannon Entropy	ShE
	27	Signal Quality Index	SQI
	28	Standard Deviation	STD
	29	Variance	
Frequency	30	Adaptive organization index	AOI
	31	Differences of the maximal spectral peak positions	
	32	Fractional spectral radius	FSR
	33	Kurtosis of the spectrum	
	34	Maximal Spectral Peak	
	35	Peak to sum ratio	
	36	Permutation entropy	
	37	Spectral Entropy	SE
	38	Spectral Powers Coefficient	
	39	Spectral purity index	SPI
	40	Summed spectral energy	
	41	The variance of the slope of the phase difference	
Time-Frequency	42	Average Energy	AE
	43	Mean Absolute Value	MAV
	44	Wavelet Power Spectrum	

PPG: photoplethysmography; AF: Atrial Fibrillation.

**Table 4:** Features found in the studies included in the systematic review.

Study	Features		Results		
	Type	ID	Sensitivity	Specificity	Accuracy
Computationally Efficient Algorithm for Photoplethysmography-Based Atrial Fibrillation Detection Using Smartphones [12].	Time Domain	8, 9, 13, 14, 18, 24, 26, 28	85–100%	99–100%	96–100%
	Frequency Domain	31, 33, 34, 35, 37, 38, 40			
Can one detect atrial fibrillation using a wrist-type photoplethysmographic device? [13].	Time Domain	6, 7, 8, 14, 15, 24, 28	98.1%	88.7%	95.9%
	Frequency Domain	30, 32, 36, 37, 39, 41			
A Deep Learning Approach to Monitoring and Detecting Atrial Fibrillation using Wearable Technology [14].	Time Domain	22, 25, 27, 28	Not reported	Not reported	91.8%
	Time-Frequency Domain	44			
Using Support Vector Machines for Atrial Fibrillation Screening [15].	Time Domain	1, 7, 15, 8, 14, 26, 29	Not reported	Not reported	97.3–99.4%
	Time-Frequency Domain	28, 42, 43			
Atrial Fibrillation Detection Using a Novel Cardiac Ambulatory Monitor Based on Photo-Plethysmography at the Wrist [16].	Time Domain	4, 16, 17	98.1%	88.7%	95.9%
Comparison between electrocardiogram and photoplethysmogram derived features for atrial fibrillation detection in free-living conditions [17].	Time Domain	4, 18, 19, 24, 25, 26	98.4%	98%	98.1%
Detection of atrial fibrillation using a photoplethysmographic earlobe sensor [18].	Time Domain	2, 5, 19, 28	94.3–100%	94.4–95.8%	NR
Monitoring of heart rate and inter-beat-intervals with wrist photoplethysmography in patients with atrial fibrillation [19].	Time Domain	10, 12, 21, 23	99%	93%	NR
Monitoring and Detecting Atrial Fibrillation using Wearable Technology [20].	Time Domain	22, 25, 27, 28	97%	94%	95%
Detection of Beat-to-Beat Intervals from Wrist Photoplethysmography in Patients with Sinus Rhythm and Atrial Fibrillation After Surgery [21].	Time Domain	10, 11, 12, 19, 23, 24, 28	Not reported	Not reported	97.49%
Motion and Noise Artifact-Resilient Atrial Fibrillation Detection using a Smartphone [22].	Time Domain	24, 26	96.67%	97.65%	97.14%
A Comparative Evaluation of Atrial Fibrillation Detection Methods in Koreans Based on Optical Recordings using a Smartphone [23].	Time Domain	24, 26	Not reported	97.52%	96.76%
Diagnostic assessment of a deep learning system for detecting atrial fibrillation in pulse waveforms [24].	Time Domain	3, 4, 18, 26, 28	84.5–96.4%	81.9–96.1%	Not reported
Smart detection of atrial fibrillation [25].	Time Domain	24, 26, 28	87.5–95%	95%	Not reported
Detection of Atrial Fibrillation Episodes Using a Wristband Device [26].	Time Domain	8, 19, 24, 25, 28	75.4%	96.3%	Not reported
Validating Features for Atrial Fibrillation Detection from Photoplethysmogram under Hospital and Free-living Conditions [27].	Time Domain	19, 24, 25, 26	69–93.9%	44.2–94.3%	68.4–83.8%

**Table 5:** The accuracy obtained by the models implemented for each group of features.

MODELS						
Accuracy						
Features	XGB Classifier	Kneighbors Classifier	Decision Tree Classifier	Random Forest Classifier	AdaBoost Classifier	Gradient Boosting Classifier
**Group 1**	98.55%	97.15%	96.66%	97.94%	98.55%	97.88%
**Group 2**	98.91%	97.45%	97.76%	98.85%	98.85%	98.24%
**Group 3**	98.97%	97.70%	96.86%	97.51%	98.85%	98.42%
**Group 4**	98.79%	97.70%	96.19%	97.51%	98.67%	98.24%
**Group 5**	98.61%	97.15%	96.61%	98.18%	98.67%	98.42%
**Features**	**GaussianNB**	**SVC**	**NuSVC**	**Linear Discriminant Analysis**	**Quadratic Discriminant Analysis**	·
**Group 1**	96.86%	50.96%	56.99%	93.81%	97.64%	·
**Group 2**	97.28%	56.92%	76.77%	95.46%	97.28%	·
**Group 3**	97.10%	55.18%	70.07%	95.34%	90.89%	·
**Group 4**	96.98%	55.30%	71.52%	95.52%	91.35%	·
**Group 5**	96.92%	50.78%	56.33%	93.81%	85.33%	·

**Table 6:** Statistical results extracted from precision.

Feature	Models					
	XGB Classifier	Kneighbors Classifier	Decision Tree Classifier	Random Forest Classifier	AdaBoost Classifier	Gradient Boosting Classifier
Media	98.76%	97.43%	96.81%	98.00%	98.72%	98.24%
Standard error	0.0008	0.0012	0.0026	0.0025	0.0006	0.0010
Median	98.79%	97.45%	96.66%	97.94%	98.67%	98.24%
Standard deviation	0.0018	0.0028	0.0058	0.0055	0.0013	0.0022
Sample variance	0.0000	0.0000	0.0000	0.0000	0.0000	0.0000
Minimum	98.55%	97.15%	96.19%	97.51%	98.55%	97.88%
Maximum	98.97%	97.70%	97.76%	98.85%	98.85%	98.42%
Count	5	5	5	5	5	5
**Feature**	**GaussianNB**	**SVC**	**NuSVC**	**Linear Discriminant Analysis**	**Quadratic Discriminant Analysis**	·
Media	97.03%	53.83%	66.34%	94.79%	92.50%	·
Standard error	0.0007	0.0125	0.0411	0.0040	0.0229	·
Median	96.98%	55.18%	70.07%	95.34%	91.35%	·
Standard deviation	0.0017	0.0279	0.0918	0.0089	0.0511	·
Sample variance	0.0000	0.0008	0.0084	0.0001	0.0026	·
Minimum	96.86%	50.78%	56.33%	93.81%	85.33%	·
Maximum	97.28%	56.92%	76.77%	95.52%	97.64%	·
Count	5	5	5	5	5	·

**Table 7:** Statistical results of the XGBClassifier and AdaBoostClassifier models.

Model	Accuracy	Sensitivity	Specificity	Precision	F Score	ROC Score
XGB Classifier	98.97%	98.43%	99.52%	99.51%	0.989677	98.97%
AdaBoost Classifier	99.15%	98.55%	99.15%	99.15%	0987249	98.85%
